# A Novel and Alternative Treatment Method for Diabetic Heel Ulceration Exposing the Calcaneus Which Is Not Suitable for Flap Surgery: Vacuum Assisted Sandwich Dermal Matrix

**DOI:** 10.1155/2015/785819

**Published:** 2015-09-21

**Authors:** Ugur A. Bingol, Can Cinar, Hakan Arslan, Muzaffer Altındas

**Affiliations:** ^1^Department of Plastic, Reconstructive and Aesthetic Surgery, Yeditepe Medical School, Yeditepe University, Devlet Yolu Ankara Caddesi No. 102-104, Kozyatağı, 34752 Istanbul, Turkey; ^2^Department of Plastic, Reconstructive and Aesthetic Surgery, Istanbul University Cerrahpasa Faculty of Medicine, 34098 Istanbul, Turkey; ^3^Private Office, 34365 Istanbul, Turkey

## Abstract

*Background*. Currently, free flaps and pedicled flaps are the first treatment choices for large heel ulcer reconstruction. However, flap reconstruction of heel ulcerations cannot be performed in all diabetics especially with concurrent severe peripheral vascular disease because of higher flap failure rate. In recent years, the use of acellular dermal matrix (ADM) has emerged as an alternative treatment option for extremity ulcers. *Methods*. We present 13 diabetic patients with a large heel ulceration exposing the calcaneus, who were not eligible for flap surgery due to the presence of only one patent artery of trifurcation. These cases were treated with the vacuum assisted sandwich dermal matrix (VASDEM) method. *Results*. None of the patients required amputation. Skin grafting was successful in ten patients. Although partial losses were observed in three patients, they were healed spontaneously without surgical interventions. During the follow-up period none of the patients developed ulceration on the treatment area. All patients maintained their preoperative ambulatory ability. *Conclusion*. VASDEM is a novel method offering opportunity for treatment before proceeding to amputation in diabetic heel ulceration exposing the calcaneus which is not suitable for flap surgery. It also has the potential to close wounds of all sizes independent of the vessel status and wound size in selected diabetic patients.

## 1. Introduction

The functional repair of heel ulcerations is one of the most important challenges faced by surgeons in the treatment of diabetic patients with heel ulcerations exposing the calcaneus and accompanying vascular disorders. Currently, free flaps and pedicled flaps are the first choices of treatment in the repair of large heel ulcerations [1]. Flap reconstruction of heel ulcerations cannot be performed in all patients with diabetes and concurrent severe peripheral vascular disease, and flap failure rates are higher in surgically treated cases [2–5]. In particular, diabetic patients with large ulcerations may face the possibility of major amputation if a suitable recipient vessel cannot be found [6]. Diabetic patients account for 40–70% of all cases undergoing lower extremity amputation [7]. This fact has prompted reconstructive surgeons to develop alternative treatment approaches.

In recent years, the use of acellular dermal matrix (ADM) has emerged as an alternative option in the treatment of selected cases with extremity ulcerations [8]. The present paper presents 13 diabetic patients with a large heel ulceration exposing the calcaneus, who were not eligible for flap surgery due to the presence of only one patent artery among the three arteries that establish trifurcation. These cases were treated with the vacuum assisted sandwich dermal matrix (VASDEM) method, which was developed to avoid amputation and allow functional closure of the wounds. This method shows the potential to close all wounds of various sizes and depths and is easily applicable due to a lack of need for microsurgery or flap surgery.

## 2. Materials and Methods

The medical records of 13 patients, who underwent heel reconstruction surgery using the VASDEM method between December 2010 and December 2014, were retrospectively reviewed after the patients had provided written informed consent. The study was approved by the Institutional Review Board. All patients had large heel ulcerations exposing the calcaneus that could not be repaired by local skin flaps. The study group consisted of patients who had severe peripheral vascular disease and only one patent vessel among the three vessels that establish the infrapopliteal trifurcation. The patients with uncontrolled systemic infection originating from the wound were not included in the study until the infection was controlled. However, the presence of local soft tissue infection or osteomyelitis was not considered an exclusion criterion for the study. All patients had type 2 diabetes and were using insulin injections, and all patients were male.

The mean age was 50.6 years (range: 39–59 years). The etiology of the wound was neuropathic heel ulceration associated with diabetes mellitus in 11 patients (84.6%), acute arterial insufficiency in one patient (7.7%), and burn in one patient (7.7%). The mean wound diameter was 5.07 cm (range: 4–8 cm). The mean duration of negative pressure wound therapy was 32.76 days (range: 24–57 days). The mean duration of follow-up was 28.69 months (range: 10–42 months). The blood glucose levels were regulated in all patients before performing reconstruction with ADM. In all patients, vascular patency in the lower extremity was examined by a vascular surgeon, and all patients were evaluated for the indication of bypass grafting, stenting, or balloon dilation using MR angiography. Vascular surgical procedures were performed before the therapy as indicated. All patients included in the study received antiplatelet therapy before and after surgery (Table 1).

### 2.1. Surgical Method

**Figure 1:**
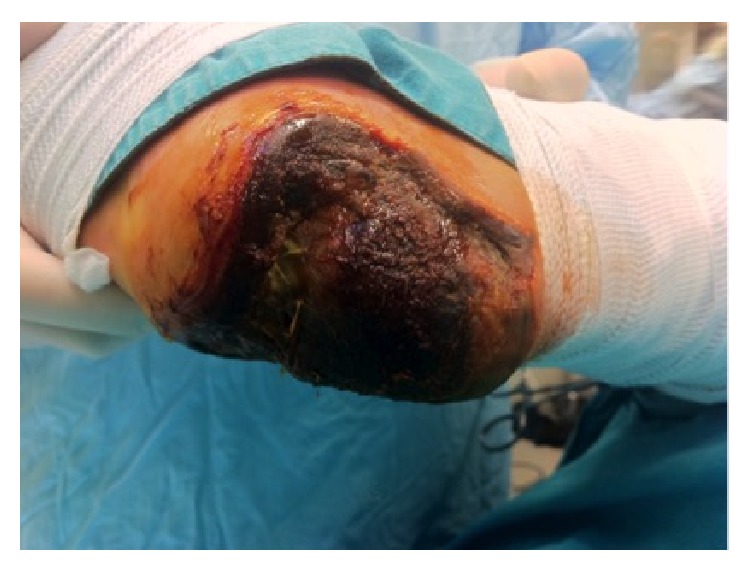
Full thickness necrosis due to acute limb ischemia (patient 1 in Table 1, right heel).

**Figure 2 fig2:**
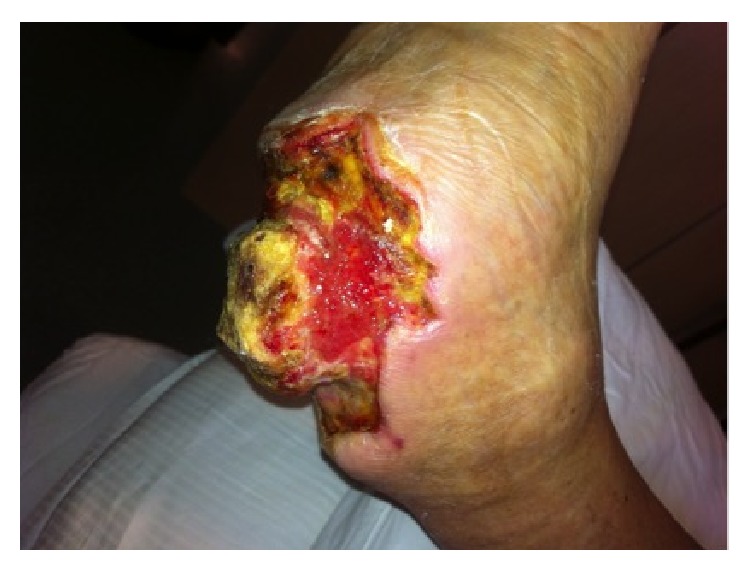
After serial wound debridements appearance of peripheral granulation tissue. Medial view.

**Figure 3 fig3:**
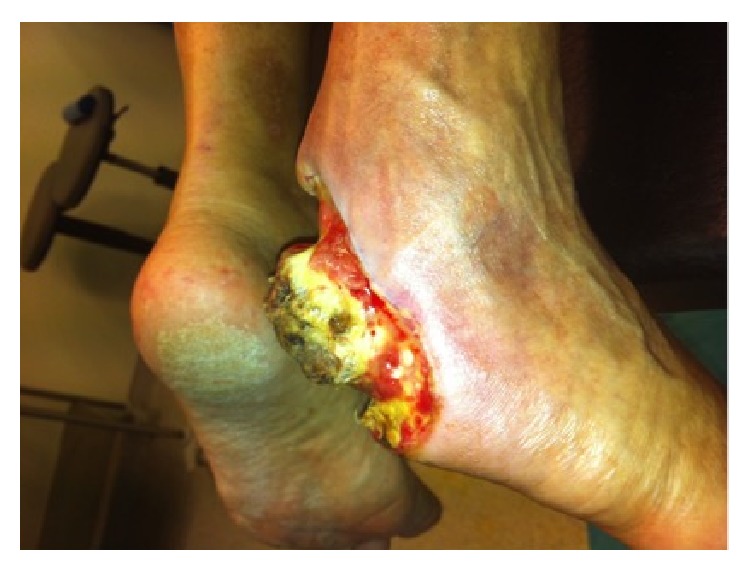
After serial wound debridements appearance of peripheral granulation tissue. Lateral view.

**Figure 4:**
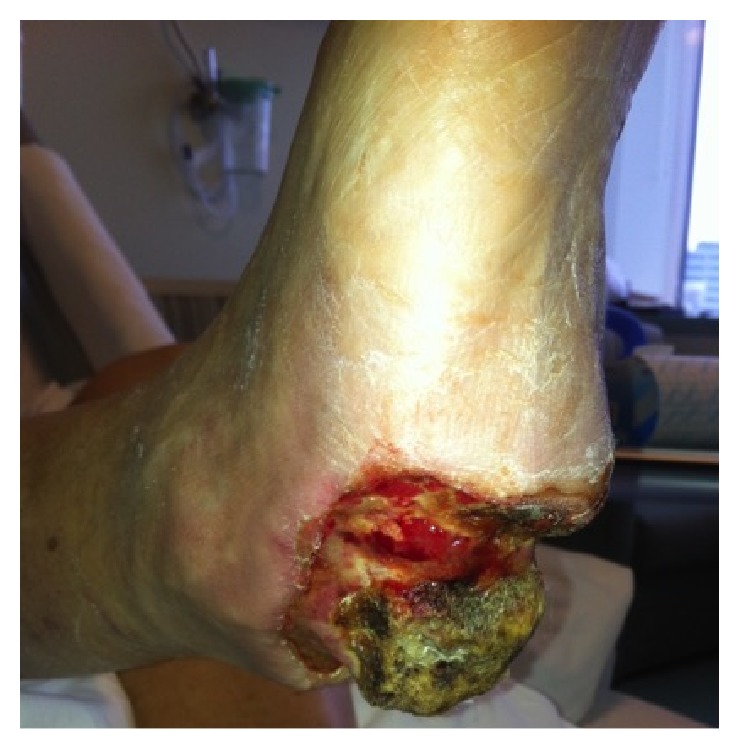
After serial wound debridements appearance of peripheral granulation tissue. Oblique view.

**Figure 5:**
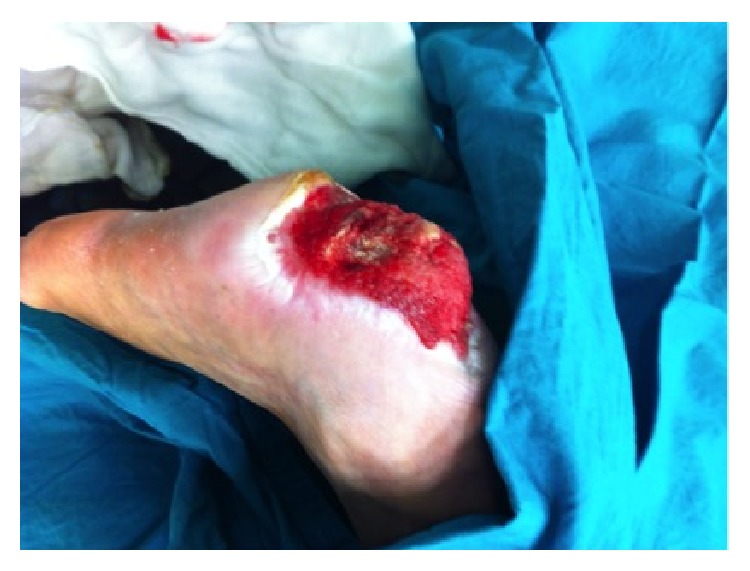
Most of the wound filled with healthy granulation tissue.

**Figure 6:**
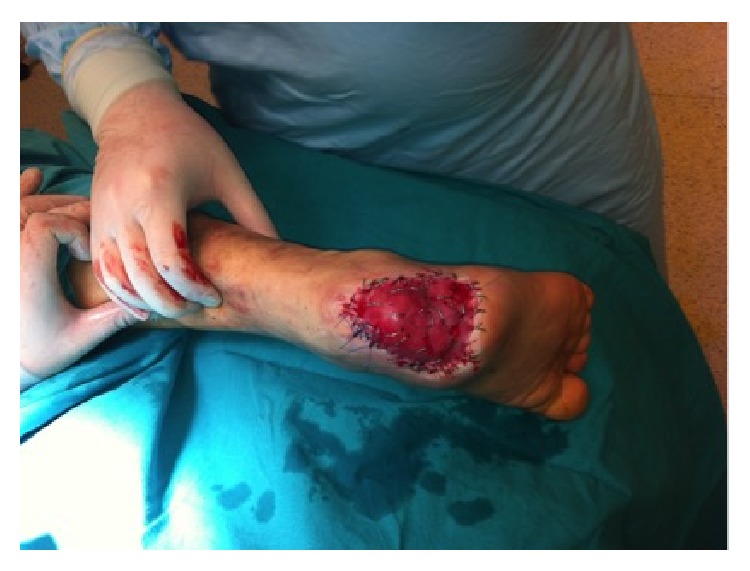
Immediate view after ADM application.

**Figure 7:**
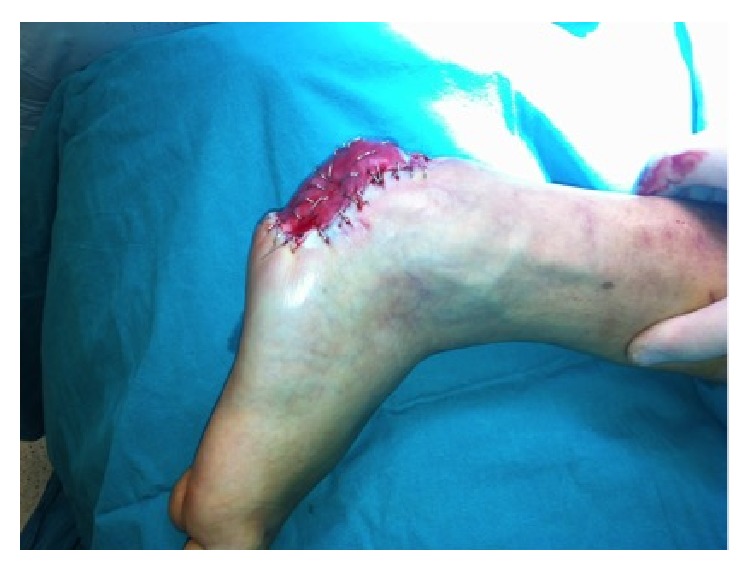
Immediate view after ADM application.

**Figure 8:**
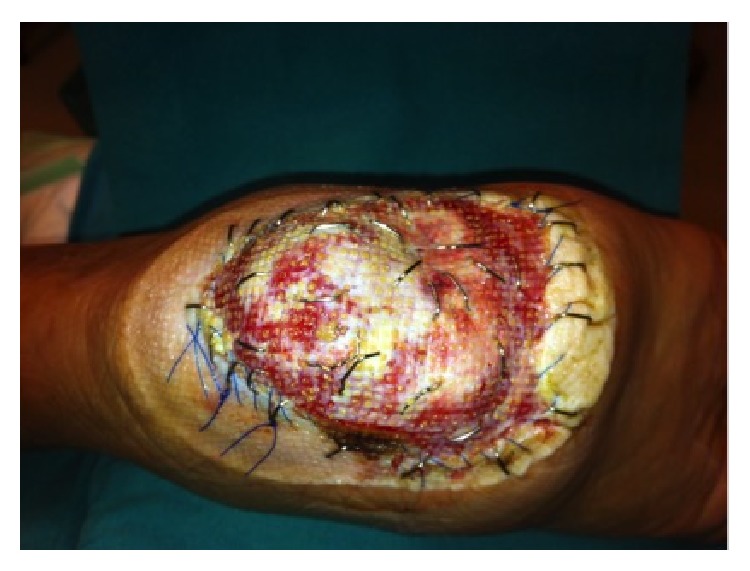
Granulation tissue formation over ADM.

**Figure 9:**
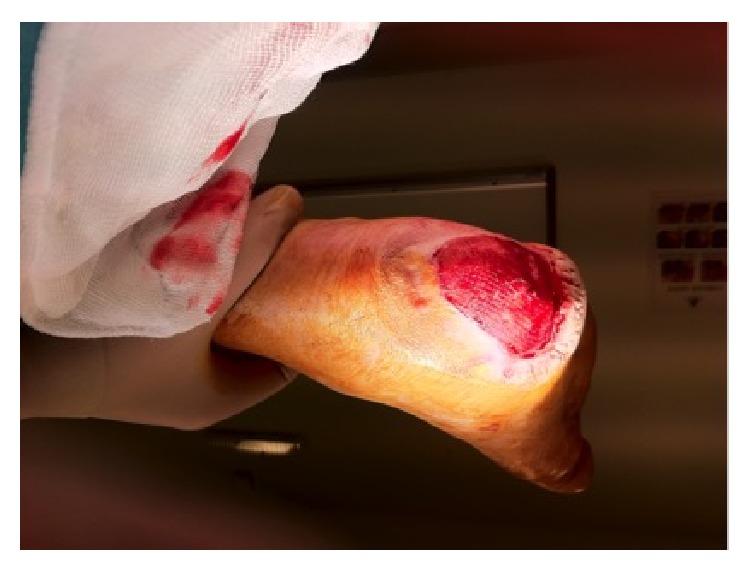
Granulation tissue formation over the second layer of ADM.

**Figure 10:**
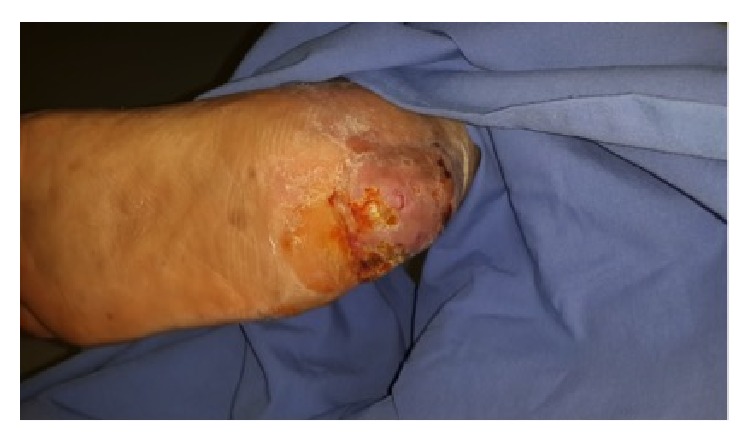
The foot at 42 months.

**Figure 11:**
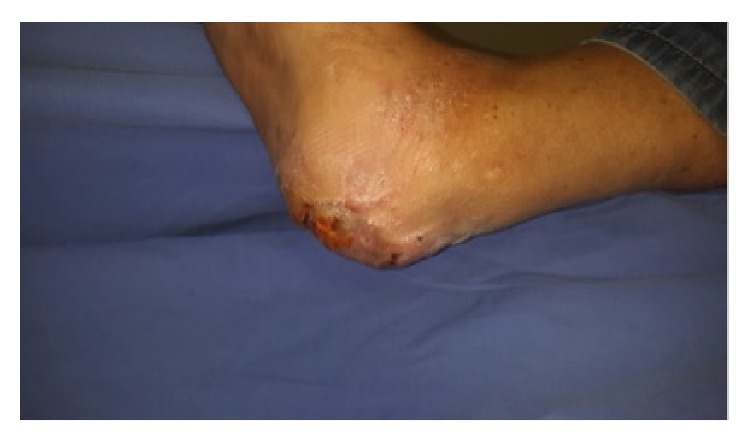
The foot at 42 months

A deep wound tissue biopsy culture was performed to determine wound infection before the procedure, and appropriate antibiotherapy was initiated as guided by the culture results. When osteomyelitis was diagnosed, bone debridement was performed in addition to soft tissue debridement (Figures 1 [Fig fig2] [Fig fig3]–4). Following the debridement, negative pressure wound therapy (NPWT) (V.A.C. KCI, San Antonio, TX, USA) was administered using 125 mmHg negative pressure and silver impregnated polyurethane foam in an intermittent manner (2/6) at 72-hour intervals. The wound site was reassessed every 72 hours, and debridement was performed for new necrotic sites. After the observation of peripheral granulation tissue, drill holes were created 5 mm apart on the exposed calcaneus using a 2mm drill in patients who did not have osteomyelitis, and NPWT was continued (Figure 5). After the wound floor had been covered with granulation tissue, BellaDerm (MTF Musculoskeletal Transplant Foundation, NJ, USA) ADM mesh was applied together with NPWT to the wound as the dermal aspect would face the calcaneus if bacterial cultures did not show an infection (Figures 6 and 7). NPWT dressings were changed every 72 hours. After ADM had been completely covered by the granulation tissue, the second layer of the dermal matrix was placed as the dermal aspect would face the previous ADM. NPWT dressings were changed every 72 hours. After the formation of granulation tissue on the second layer of ADM, the wound was closed with split-thickness skin graft (0.4 mm) and NPWT (Figures 8 and 9). The dressings (NPWT) were controlled on day 6. If the grafts appeared normal, the operative site was opened and graft maintenance was performed every 72 hours. Lower extremity splints were not removed before day 10 in the postoperative period. After postoperative day 10, active range of motion (ROM) exercises were initiated for the ankle joint. The patients were mobilized with two walking aids without weight bearing for four weeks. Full-weight bearing was allowed at the end of the first month depending on the recovery status. The patients were trained on the application of dexpanthenol- and chlorhexidine dihydrochloride-containing (Bepanthen plus cream, Bayer) creams twice a day to avoid dryness and moisturize the grafted skin site (Figures 10 and 11).

## 3. Results

**Figure 12:**
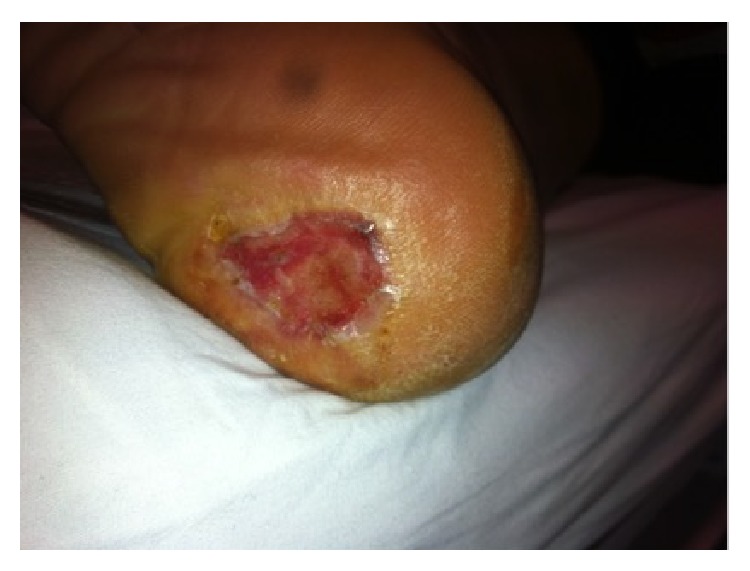
The view of the wound caused by thermal injury seven months after the operation. ADM exposure (patient 8 in Table 1, right heel).

**Figure 13:**
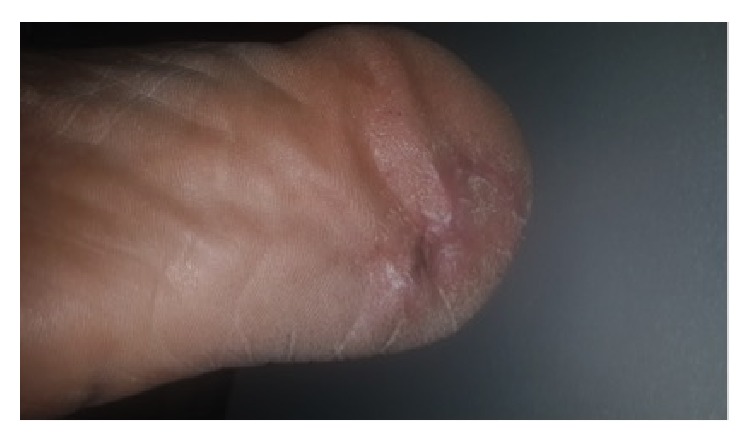
After the epithelialization.

None of the patients treated with ADM required amputation. None of the patients developed a wound site infection in the postoperative period. The mean duration for the formation of granulation tissue was 12 days for the wound floor (range: 9–18 days) and nine days for the first and second layers of ADM (range: 6–12 days). None of the patients experienced loss of ADM. None of the patients developed a hematoma or seroma. Skin grafting was successful in ten patients (77%) and partial losses were observed in three patients (23%). However, these losses showed recovery with dressings without requiring additional interventions. None of the patients developed ulceration at the skin site grafted with ADM. Four patients (30.77%) developed several wounds at the junction of the ADM and normal skin, and all recovered spontaneously with local wound care. This complication did not prevent mobilization of the patients. Of the 13 patients, four (30.8%) required orthopedic shoes, whereas the remaining nine (69.2%) patients maintained their lives with normal shoes. One of the patients who required orthopedic shoes developed a plantar flexion deformity associated with the shortening of the Achilles tendon. This patient was also using orthotics. One patient sustained a burn injury at the operation site seven months after the operation due to contact with a hot object. The superficial second-degree burn injury showed spontaneous epithelialization with wound dressing within ten days (Figures 12 and 13).

## 4. Discussion

Foot ulcers are observed in 85% of diabetic patients and are the most important causes of lower extremity amputations [9]. Foot ulcers heralding extremity amputation are often the result of peripheral vascular disease, neuropathy, or an infection, all of which may accompany diabetes [10, 11]. Heel ulcerations larger than 4 cm are the most challenging diabetic foot ulcers in terms of treatment [12, 13], and these wounds often result in below-the-knee amputation due to the presence of accompanying factors [14]. Studies have begun to appear in the literature implicating heel ulcerations larger than 4 cm as an independent risk factor for the loss of extremity [15]. Diabetes and accompanying factors that cause heel ulcerations also constitute an important obstacle for the reconstruction of these wounds.

“Replacing like with like” is one of the most important principles in reconstructive surgery. A thinner tissue that is similar to the heel is found in the palms of the hands; however, the hand is so precious that it cannot be used as a donor site for the repair of heel defects. Local flaps, island flaps, and local muscle flaps are the most appropriate options for the repair of small defects in the heel (<3 cm) [1]. Free flaps or pedicled flaps are other options for the repair of larger defects.

There is still debate over the use of free flaps or pedicled flaps in the reconstruction of large heel ulcerations occurring in diabetic patients with concurrent peripheral vessel disease. Sacrificing a vessel that supplies the entire leg for the sake of closing the wound or stealing from the flow of this vessel (steal phenomenon) can result in critical ischemia distal to the anastomosis [6, 16, 17]. Flap options can be justified only in specific conditions in diabetic patients with acute or chronic severe peripheral vascular disease [18]. The goal is to develop alternative treatment approaches for patients who are not eligible for flap reconstruction [19, 20].

The selection of a recipient vessel, the presence of appropriate anastomosis segments, and vessel perfusion are the most important factors affecting the success of free flaps in diabetic patients [21]. However, the presence of severe peripheral vascular disease in most diabetic patients makes it challenging for the surgeons to perform a free flap. Some authors suggest that there is no contraindication for free flaps with the exclusion of “no flow” conditions in the foot vessels in diabetic patients, and a success rate close to that in nondiabetic patients can be achieved with free flap techniques if “super micro surgery” techniques are used [21]. However, most surgeons may abstain from using free flaps in patients with one patent vessel due to the high risk of free flap failure [2]. Additionally, the use of the free flap is also contraindicated in diabetic patients undergoing dialysis due to kidney disease [6].

The diabetic patient group in the present study consisted of patients with severe peripheral vascular disease and large heel ulceration. These patients were not eligible for free or pedicled flaps because only one of the three vessels that constitute the trifurcation was patent.

Most importantly an ideal tissue or material for use in the repair of large heel defects is thin and stable enough to allow shoes to be worn. The tissue or material must resist trauma and shearing forces and should not easily become ulcerated; it must be resistant to ischemia and must be large enough to cover wounds of all sizes. However, donor site morbidity must also be minimal. Because ADM is traditionally used in the treatment of burn injuries, there is limited data on the use of ADM in the treatment of chronic wounds. BellaDerm is a human-derived ADM. The most prominent advantage of human-derived ADM over animal-derived ADM is that it elicits a weaker immune response and offers a safer profile for potential infections by prions. ADM integrates with the neighboring tissues and becomes a part of the host. The ADM is resistant to trauma, and it can be sutured, folded, cut, and easily shaped into rolls, similar to normal tissues. Donor site morbidity is not applicable for ADM.

The integration of ADM into the recipient site is gradual, as in other grafts involving imbibition, fibroblast migration, neovascularization, and maturation [22]. The ADMs receive nutrients via diffusion and do not require a rich blood supply.

The loss of ADM is inevitable as it is not sufficiently attached to the recipient zone. It is not a new practice to apply NPWT dressing with dermal matrices instead of classical bolster dressing [23]. NPWT dressing reduces shearing forces by applying equal negative pressure to the wound and the graft [24]. NPWT dressings have been shown to increase skin graft success compared to classical bolster dressing [25, 26]. Although all patients in the present study were using medications that caused a predisposition to bleeding, the aim of using NPWT dressing was to increase graft success by decreasing the risk of hematoma formation.

The use of NPWT dressing alone is known to have favorable effects in the treatment of diabetic foot ulcers [27]. Working synergistically, the use of ADM and NPWT dressing aimed to preclude problems that would arise during wound healing.

Under normal conditions, the thickness of heel skin varies between 0.9 and 1.3 mm [28]. ADM strictly adheres to the calcaneus in a uniform manner and is still very thin, allowing for skin grafting. Because diabetic patients are more vulnerable to the development of ulcers, the tissue thickness is augmented by placing two layers of ADM instead of a single layer to protect the exposed calcaneus from small traumas and increase the potential for spontaneous recovery if ulcers develop. By applying two layers of BellaDerm (thick type, thickness: 0.8–1.7 mm), the tissue thickness was augmented to 1.6–3.4 mm, which does not include the thickness of the granulation tissue or skin graft. The tissue can resist shearing forces due to the strong adherence between the calcaneus and ADM, strong adherence between the two ADMs, and strong adherence between ADM and the skin graft.

Although it theoretically seems intuitive that plantar sensation is required for the prevention of ulcers, this subject remains controversial. In most cases, a deep protective sensation seems sufficient for the protection of flaps [29–31]. The deep protective sensation, which seems to have a protective role for the flaps, can also contribute to the reduction in the development of ulcers in ADMs, which are tightly fixed to the bone and are thinner than the flaps.

Another advantage of this method is that complex reconstruction can be postponed to a later date in trauma patients who are not eligible for free or pedicled flaps in the acute phase, independent of the conditions of the vessels.

The current series of patients constitutes a homogeneous group in terms of location and extent of the wound site and vascular status. This is the first series in the literature in which two layers of ADMs were used.

The most important drawbacks associated with this method include the high costs of NPWT and ADMs, the dependence of the patient on NPWT dressing, even for a short period of time, and the duration of the therapy. These methods are currently under development and available data are not sufficient to calculate the costs.

## 5. Conclusion

Diabetic patients with severe peripheral vascular disease and heel ulceration face the risk of amputation. VASDEM is a new method offering an opportunity for treatment before resorting to amputation that has the potential to close wounds of all sizes independent of the vessel status and wound size in selected diabetic patients who require complex reconstruction of the diabetic heel ulcerations.

## Figures and Tables

**Table 1:** Characteristics of patients with heel defects managed with VASDEM.

Number	Sex	Age	Type of wound/ulcer	Defect size/cm	Follow-up/month	NPWT/days	Special shoe	Vascular procedures		
								Bypass graft	Vascular stent	Balloon dilatation
1	M	39	Acute arterial insufficiency	8	42	57	Yes	No	Yes	Yes
2	M	48	Neuropathic	4	36	27	No	No	Yes	No
3	M	46	Neuropathic	5	34	29	No	No	Yes	Yes
4	M	47	Neuropathic	5	40	31	Yes	No	Yes	Yes
5	M	48	Burn	4	26	30	No	No	Yes	No
6	M	59	Neuropathic	6	34	36	No	No	Yes	Yes
7	M	54	Neuropathic	5	26	29	No	No	Yes	Yes
8	M	47	Neuropathic	4	32	24	No	No	Yes	No
9	M	51	Neuropathic	6	23	35	Yes	No	Yes	Yes
10	M	55	Neuropathic	4	17	33	No	No	Yes	No
11	M	53	Neuropathic	5	31	35	No	No	Yes	No
12	M	56	Neuropathic	6	22	34	Yes	No	Yes	Yes
13	M	55	Neuropathic	4	10	26	No	No	Yes	No
